# A Tiny RNA that Packs a Big Punch: The Critical Role of a Viral miR-155 Ortholog in Lymphomagenesis in Marek’s Disease

**DOI:** 10.3389/fmicb.2017.01169

**Published:** 2017-06-26

**Authors:** Guoqing Zhuang, Aijun Sun, Man Teng, Jun Luo

**Affiliations:** ^1^Department of Veterinary Pathobiology, College of Veterinary Medicine & Biomedical Sciences, Texas A&M University, College StationTX, United States; ^2^Key Laboratory of Animal Immunology of the Ministry of Agriculture, Henan Provincial Key Laboratory of Animal Immunology, Henan Academy of Agricultural SciencesZhengzhou, China; ^3^College of Animal Science and Technology, Henan University of Science and TechnologyLuoyang, China

**Keywords:** herpesvirus, Marek’s disease virus, GaHV2, miR-155, miR-M4-5p, pathogenesis, tumorigenesis

## Abstract

MicroRNAs (miRNAs) are small non-coding RNAs that have been identified in animals, plants, and viruses. These small RNAs play important roles in post-transcriptional regulation of various cellular processes, including development, differentiation, and all aspects of cancer biology. Rapid-onset T-cell lymphoma of chickens, namely Marek’s disease (MD), induced by *Gallid alphaherpesvirus 2* (GaHV2), could provide an ideal natural animal model for herpesvirus-related cancer research. GaHV2 encodes 26 mature miRNAs derived from 14 precursors assembled in three distinct gene clusters in the viral genome. One of the most highly expressed GaHV2 miRNAs, miR-M4-5p, shows high sequence similarity to the cellular miR-155 and the miR-K12-11 encoded by Kaposi’s sarcoma-associated herpesvirus, particularly in the miRNA “seed region.” As with miR-K12-11, miR-M4-5p shares a common set of host and viral target genes with miR-155, suggesting that they may target the same regulatory cellular networks; however, differences in regulatory function between miR-155 and miR-M4-5p may distinguish non-viral and viral mediated tumorigenesis. In this review, we focus on the functions of miR-M4-5p as the viral ortholog of miR-155 to explore how the virus mimics a host pathway to benefit the viral life cycle and trigger virus-induced tumorigenesis.

## Introduction

MicroRNAs (miRNAs) are regulatory non-coding RNAs of approximately 22–24 nt expressed in multicellular organisms and viruses (Kozomara and Griffiths-Jones, 2011). In general, miRNAs regulate post-transcriptional processes through silencing gene expression by binding to the 3′ untranslated region (3′UTR) of mRNA transcripts (Bartel, 2009). miRNAs are involved either in maintaining physiological homeostasis or in facilitating pathogenesis, including virus infection (Bartel, 2009; Skalsky and Cullen, 2010). More than 450 virus-encoded miRNAs have been identified since they were first discovered in 2004 (Pfeffer et al., 2004). The majority of viral miRNAs are encoded by herpesviruses, such as herpes simplex virus (HSV) and human oncogenic Epstein-Barr virus (EBV), Kaposi’s sarcoma-associated herpesvirus (KSHV), and the avian *Gallid alphaherpesvirus 2* (GaHV2), highlighting the possible important roles of miRNAs in viral pathogenesis in diverse hosts (Skalsky and Cullen, 2010). Although the detailed mechanisms of EBV- and KSHV-associated disease are unclear, the recently discovered viral miRNAs are potentially powerful determinants of viral pathogenesis (Gottwein and Cullen, 2008; Skalsky and Cullen, 2010); however, a lack of suitable natural animal models has limited the direct functional analyses of viral miRNAs *in vivo* (Kincaid and Sullivan, 2012).

As an oncogenic *Alphaherpesvirus*, GaHV2 causes immunosuppression, neurological disease, and rapid-onset T-cell lymphomas in chickens known as Marek’s disease (MD) (Calnek, 2001), which has been proposed as an ideal animal model for herpes virus-related cancer research (Osterrieder et al., 2006). Previous studies have characterized the functions of some GaHV2-specific genes *in vitro* and/or *in vivo* (Osterrieder et al., 2006); however, a comprehensive understanding of the molecular mechanisms involved in GaHV2 pathogenesis is lacking. The recent discovery of viral miRNAs encoded in the GaHV2 genome raises the possibility that these tiny non-coding RNAs may be significant for viral pathogenesis, particularly in relation to the virus-induced tumorigenesis (Luo et al., 2010; Yao and Nair, 2014). Interestingly, one GaHV2-encoded miRNA, miR-M4-5p, is a functional miR-155 ortholog (Zhao et al., 2009). Moreover, the “seed region” sequences of miR-M4-5p exhibit high homology to the KSHV-encoded miR-K12-11 (Parnas et al., 2014), suggesting the same, or similar, viral infectious strategies have developed during virus evolution across various viral species. In this review, we focus on relevant research findings that highlight the critical roles of miR-M4-5p in the rapid induction of MD lymphoma.

## Overview Of miRNA Biogenesis

In miRNA biogenesis (Bartel, 2004; Cullen, 2004; Skalsky and Cullen, 2010), the majority of cellular or viral genomic sequences are first transcribed as primary miRNA (pri-miRNA) in the nucleus by RNA Polymerase II. Subsequently, the RNase III enzyme, Drosha, in concert with the RNA-binding protein, DGCR8, excise pri-miRNA to an approximately 60-nt stem-loop precursor miRNA (pre-miRNA). The pre-miRNA is transferred from the nucleus to the cytoplasm by the Exportin-5 protein directed pathway. In the cytoplasm, the RNase enzyme, Dicer, cleaves the pre-miRNA into a double-stranded of mature miRNA. One of the strands (guide strand), combined with the RNA-induced silencing complex (RISC), orchestrates the regulatory role of targeting and inducing mRNA degradation and/or translational inhibition (Bartel, 2004). The other strand, known as the passenger strand or star miRNA (miRNA^∗^), is generally thought to be degraded; however, both guide and passenger strands are biofunctional in some cases (Czech et al., 2009; Okamura et al., 2009). Aside from the canonical pathway, there is an alternative, non-canonical generation of pre-miRNA by splicing and disbranching of short hairpin introns, rather than Drosha enzyme processing, known as “mirtrons” (Okamura et al., 2007; Ruby et al., 2007). Most viral miRNAs are processed and matured through the canonical pathway (Cullen, 2011) whereas viral mirtrons have rarely been identified (Rasschaert et al., 2016).

In miRNA-induced gene silencing, the miRNA serves to guide RISC and in binding to target mRNA, whereas the Argonaut (AGO) proteins function as translational inhibition effectors (Flores et al., 2014; Jonas and Izaurralde, 2015). At the 5′-end of the miRNA, 2–7 nt sequences termed as “seed region” are crucial for mRNA target recognition and miRNA-mediated repression function, which usually matches the 3′UTR of the target mRNA. Evolutionary conservation of miRNAs and their 3′UTR binding sites facilitates computational prediction and experimental identification of authentic miRNA targets (Bartel, 2009). Computational analysis suggests that a single miRNA may target hundreds of genes, whereas one gene may be regulated by multiple miRNAs. Over 60% of human protein-coding genes contain at least one conserved miRNA binding site (Friedman et al., 2009), suggesting an important role for miRNA-mediated gene expression regulation.

## miR-155 and Its Viral Ortholog

miR-155 was initially identified as a B-cell integration cluster (*bic*) gene, which is activated by promoter insertion at a retroviral integration site in avian leukosis virus (ALV)-induced lymphomas (Clurman and Hayward, 1989; Rodriguez et al., 2007). miR-155 is highly conserved in humans, mice, and chickens, particularly the seed region, primarily expressed in lymphocytes of the thymus and spleen, and has regulatory functions in the hematopoietic and immune systems (Rodriguez et al., 2007). Over-expression of miR-155 is related to B cell lymphomas and solid tumors (Tili et al., 2013).

miR-155 is also involved in viral pathogenesis, particularly that of the tumor-related herpesviruses. EBV is a human malignancy related gammaherpesvirus, which induces B-cell lymphomas, including Hodgkin’s and Burkitt’s lymphomas and other types of human cancer (Young and Rickinson, 2004). EBV encodes 25 pre-miRNAs and 44 mature miRNAs (miRBase v21), which play important regulatory roles in virus-induced tumorigenesis (Skalsky and Cullen, 2010; Qiu et al., 2015). Interestingly, EBV-induced cell proliferation, as well as virus latency and reactivation, are associated with elevated miR-155 expression levels (Yin et al., 2008; Linnstaedt et al., 2010; Forte and Luftig, 2011). Importantly, it has been clearly shown that EBV-induced overexpression of cellular miR-155 is essential for transformation by EBV (Linnstaedt et al., 2010).

Kaposi’s sarcoma-associated herpesvirus is another causative agent of human diseases, particularly Kaposi’s sarcoma, primary effusion lymphoma (PEL), and multicentric Castleman’s disease (Ganem, 2006). KSHV encodes 13 pre-miRNAs and 25 mature miRNAs (miRBase v21), which also have important regulatory roles in viral pathogenesis (Gottwein and Cullen, 2008; Kincaid and Sullivan, 2012). It is of great interest that the KSHV-encoded miR-K12-11 functions as a viral ortholog of miR-155 (Gottwein et al., 2007; Skalsky et al., 2007). Both miR-K12-11 and miR-155 are associated with human lymphoma and share a common set of mRNA targets (Gottwein et al., 2007; Skalsky et al., 2007; McClure and Sullivan, 2008). The potentiality of miR-K12-11 compensating for the function of miR-155 has been proved in humanized and transgenic miR-155 knockout mice; however, this is difficult to be confirmed because of the lack of a natural animal model for KSHV infection (Boss et al., 2011; Sin et al., 2013).

## Viral miRNAs Encoded By GaHv2

The MD associated avian herpesviruses, were previously classified into serotype 1 (MDV-1), serotype 2 (MDV-2) and herpesvirus of Turkeys (HVT), have recently been reclassified as GaHV-2, *G. alphaherpesvirus 3* (GaHV-3), and *Meleagrid alphaherpesvirus 1* (MeHV1), respectively^1^. Similar to the other double-stranded DNA herpesviruses (Pfeffer et al., 2004, 2005), all three virus serotypes are confirmed to encode miRNAs (Burnside et al., 2006, 2008; Yao et al., 2007, 2008, 2009; Waidner et al., 2009).

GaHV2-encoded miRNAs were identified in virus-infected chicken embryo fibroblasts (CEFs) and virus-transformed T-lymphoma cells (Burnside et al., 2006, 2008; Yao et al., 2008). Three distinct miRNA clusters in the GaHV2 genome has been confirmed. The first cluster, miR-M9-M4, referred to as the Meq-cluster (Burnside et al., 2006; Yao et al., 2008), is located upstream of the *meq* oncogene and includes six pre-miRNAs (miR-M9, miR-M5, miR-M12, miR-M3, miR-M2, and miR-M4 in order). Cluster miR-M11-M1, namely the Mid-cluster (Luo et al., 2010), is located downstream of *meq* and includes three pre-miRNAs (miR-M11, miR-M31, and miR-M1). A third cluster, miR-M8-M10, referred to as the LAT-cluster (Burnside et al., 2006; Yao et al., 2008), is found within the first intron of the GaHV2-encoded 10 kb LAT (latency-associated transcript), and includes five pre-miRNAs (miR-M8, miR-M13, miR-M6, miR-M7, and miR-M10). The sequences of GaHV2 miRNAs from a collection of field and reference strains with various levels of virulence were compared and found to be highly conserved; however, miRNAs expressed from the Meq and Mid- clusters were detected at higher levels in lymphomas caused by a very virulent plus (vv+) strain than in those caused by a less virulent strain. In contrast, expression levels of the miRNAs from the LAT-cluster were equivalent in tumors produced by vv and vv+ strains (Morgan et al., 2008).

Zhao et al. (2011) reported that deletion of the Meq-cluster significantly attenuated the oncogenicity of virulent GaHV2, strongly implicating this cluster as a key regulator of viral pathogenesis. A similar deletion of the Meq-cluster in the very virulent GX0101 strain significantly decreased, rather than abolished, its oncogenicity (Yu et al., 2014). These results suggest that the Meq-cluster is an important, but dispensable, regulator of the development of MD lymphomas. Interestingly, deletion of individual miRNAs of the Meq-cluster produced no effect on virus replication; however, each of the mutants was associated with reduced mortality and gross tumor incidence after infection relative to the parental virus (Zhao et al., 2011; Yu et al., 2014; Teng et al., 2015). The individual miRNA mutants produced variable effects on mortality and gross tumor production in infected chickens, suggesting that they may be involved in different cellular signaling pathways. For example, miR-M3-5p targets and down-regulates the expression of SMAD2, a critical component of the transforming growth factor beta (TGF-β) signaling pathway (Xu et al., 2011). Through suppression of SMAD2, miR-M3-5p significantly promotes cell survival and blocks cisplatin-induced apoptosis, suggesting that viruses may encode miRNAs to directly target cellular factors involved in antiviral processes, including apoptosis, thus proactively creating a cellular environment beneficial to viral latency and oncogenesis (Xu et al., 2011). miR-M4-5p is another member of this cluster which has a specific role in GaHV2 pathogenesis and will be described in more detail below.

The transcription of Mid- and Meq-clusters is driven by a single promoter (prmiRM9M4) during latent phase, while only the Meq-cluster is transcribed during the lytic phase by prmiRM9M4 (Coupeau et al., 2012), implying distinct roles of the Mid-cluster in GaHV2 infection. The Mid-cluster and the associated individual miRNAs are dispensable for GaHV2 replication. However, deletion of miR-M31 decreased the mortality and gross tumor incidence of infected chickens (Teng et al., 2017). miR-221 targets and suppresses the expression of a key cell cycle regulatory protein p27^Kip1^, which is involved in the induction and progression of T-cell lymphomas (Lambeth et al., 2009). Similar gene regulatory networks may be hijacked by miR-M31-3p, which shares a conserved seed homolog with miR-221 (Morgan et al., 2008). In contrast, deletion of miR-M11 unexpectedly increased viral pathogenicity, and oncogenicity may be partially induced by one member of the mature miRNAs, miR-M11-5p, which targets and suppresses *meq* expression. Overall, these results suggest that miR-M31-3p may act as an oncogene, while miR-M11-5p may be a potential tumor suppressor during GaHV2 infection (Teng et al., 2017).

The miRNAs in the LAT-cluster are similarly transcribed in various virulent GaHV2-induced tumors (Morgan et al., 2008); however, they exhibit different temporal and spatial transcription profiles during each infectious phase (Luo et al., 2011; Zhao et al., 2015). Interestingly, the GaHV2 immediate-early (IE) genes, *ICP4* and *ICP27*, are putative targets of miR-M7-5p (Strassheim et al., 2012), a member of the LAT-cluster. As a transactivator, ICP4 regulates transcription of early (E) and late (L) genes during virus infection (Pratt et al., 1994; Kato et al., 2002), and it is also necessary for transformation maintenance (Xie et al., 1996), while ICP27 interferes with viral and cellular post-transcriptional splicing (Amor et al., 2011). By targeting these two IE genes, miR-M7-5p may contribute to the establishment and maintenance of latency. These results suggest that, as with other herpesviruses, IE gene-specific miRNAs are common regulatory factors during herpesvirus cytolytic and latent infection (Murphy et al., 2008; Bellare and Ganem, 2009).

## miR-M4-5p Functions As A Viral miR-155 Ortholog

**FIGURE 1:**
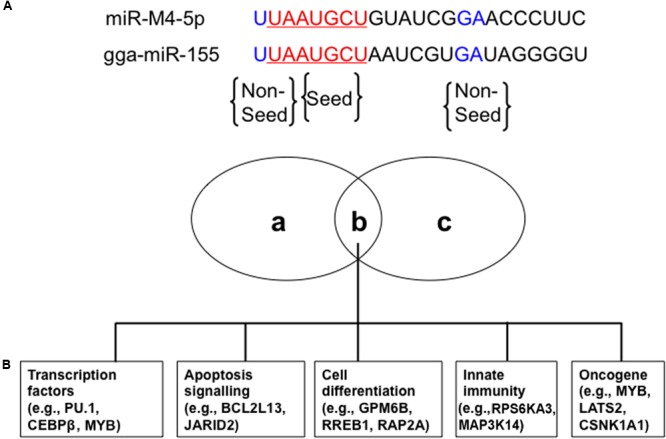
Conserved cellular mRNA targets of GaHV2-encoded orthologs of cellular miR-155. **(A)** miR-M4-5p seed (red) and non-seed (blue) sequences homologous with chicken gga-miR-155. **(B)** Venn diagram of cellular mRNA targets of both miR-M4-5p and gga-miR-155 involved in different cellular processes with potential relevance to tumorigenesis: (a) and (c) represent miR-M4-5p or gga-miR-155 specific cellular mRNA targets, while (b) represents the shared set of cellular mRNA targets. Adapted from Parnas et al. (2014).

miR-M4-5p, which is only encoded by GaHV2, rather than by GaHV3 or MeHV1, has been previously characterized as a viral miR-155 ortholog, sharing an identical seed region with both miR-155 and the KSHV-encoded, miR-K12-11 (Morgan et al., 2008; Zhao et al., 2009; **Figure 1A**). Using an miRNA target prediction algorithm (Bartel, 2009), candidate cellular mRNA target sites were identified and it was subsequently confirmed that miR-M4-5p and miR-155 down-regulate the expression of a set of shared mRNA targets, including *PU.1, CEBPβ, BCL2L13, HIVEP2, PDCD6, Myb, GPM6B, RREB1*, and *MAP3K7IP2*. These targets were confirmed using dual luciferase reporter assays (DLRA) and qRT-PCR analysis, as well as western blotting (Zhao et al., 2009; Muylkens et al., 2010; **Figure 1B**). In addition, Parnas et al. (2014) performed photoactivatable ribonucleoside-enhanced cross-linking and immunoprecipitation (PAR-CLIP) analysis of RISC binding sites in the GaHV2-transformed MSB-1 cell line to identify targets of miR-M4-5p, and then compared to those of miR-155 and miR-K12-11 similarly identified in EBV-transformed lymphoblastoid cell lines (LCLs) or a KSHV-transformed PEL cell line. The data demonstrated that nine mRNA targets (*C1orf103, CSNK1A1, LATS2, MAP3K14, MYB, NR1D2, RORA, RPS6KA3*, and *WEE1*) could be detected in all three cell lines, while four additional mRNA targets (*FCHSD2, JARID2, PBEF1*, and *RAP2A*) were only identified in the MSB-1 and LCLs cell lines (Parnas et al., 2014; **Figure 1B**). These potential mRNA targets were further confirmed by DLRA, the results of which showed that targeting of the 3′UTRs of these genes via the seed regions of miR-155 family members is conserved between humans and chickens, although the data did not demonstrate a statistically significant difference in response to miR-M4-5p and/or miR-155 expression in all cases (Parnas et al., 2014). Among these mRNA targets, *WEE1* encodes a kinase that blocks cell-cycle progression and has been associated with inflammation and cancer (Egeland et al., 2016). *JARID2* also encodes a cell cycle regulator which is part of a histone methyltransferase complex (Bolisetty et al., 2009). The miR-155/JARID2 axis could regulate cell apoptosis and differentiation in acute myeloid leukemia and abnormal megakaryopoiesis (Norfo et al., 2014; Palma et al., 2014). Three independent research groups identified MYB, a transcription factor involved in the regulation of hematopoiesis and tumorigenesis, as a target for miR-M4-5p, miR-K12-11, and miR-155 (Muylkens et al., 2010; Zhao et al., 2011; Parnas et al., 2014). It is clear that miR-155 could have an important regulatory role in many biological pathways of significance in oncogenesis, hence further investigation is needed to define the *in vivo* mRNA targets of this miRNA and its viral orthologs.

In recent years, bacterial artificial chromosomes (BACs) and mutagenesis techniques have greatly facilitated the introduction of mutations into viral genomes to study GaHV2 gene functions during pathogenesis (Schumacher et al., 2000). Using an infectious BAC clone, mutant viruses with deletions, or two-nucleotide mutations in the miR-M4-5p seed region have been constructed and characterized (Zhao et al., 2011). These mutations had no effect on virus replication; however, compared with the parental oncogenic GaHV2 virus, the mutations abolished its oncogenicity. Interestingly, MD incidence was restored when the viral pre-miRNA, miR-M4, was replaced by gga-miR-155, although tumor induction was slow and detected later than in chickens infected by parental and revertant viruses (Zhao et al., 2011). These observations suggest that miR-M4-5p may be an oncogenic miRNA, the functions of which could be partially restored by miR-155 because the two molecules regulate a common set of mRNA targets through their conserved seed region.

**FIGURE 2:**
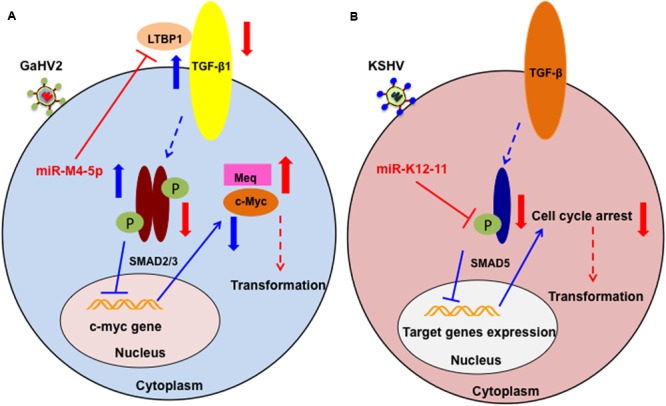
Schematic of the potential roles of miR-M4-5p and miR-K12-11 in regulating the TGF-β signaling pathway and promoting tumorigenesis. **(A)** In normal cell metabolism (blue arrows), c-Myc is down regulated by the phosphorylated SMAD2/3 complex, which is induced by LTBP1 binding to its receptor. During GaHV2 infection (red arrows), miR-M4-5p suppresses the expression levels of LTBP1, leading to reduction of the active SMAD2/3 complex, inducing increased expression of c-Myc. The potential combination of c-Myc and the GaHV2-specific oncoprotein, MEQ, promotes cellular transformation. **(B)** In normal cell metabolism (blue arrows), activated SMAD5 inhibits target gene expression. During KSHV infection (red arrows), miR-K12-11 inhibits the expression of SMAD5. Through abolishing the TGF-β signaling pathway, miR-K12-11 blocks cell cycle arrest to promote cellular transformation. **(A,B)** are adapted from Chi et al. (2015) and Liu et al. (2012), respectively.

As described above, miR-M4-5p is critical for GaHV2 oncogenicity, and has multiple candidate mRNA targets; however, the details of its precise mechanism of action remain incompletely defined. We have recently identified latent TGF-β binding protein 1 (*LTBP1*) as a bona fide host mRNA target (Chi et al., 2015). Our data demonstrated that inhibition of *LTBP1* expression by miR-M4-5p induced a significant decrease of TGF-β1 secretion and activation, following a significant increase in the expression of c-Myc, a well-known oncogene. It has also been reported that c-Myc regulation in the TGF-β signaling pathway is SMAD-responsive (Yagi et al., 2002). Thus, our data suggest another mechanism by which GaHV2 triggers c-Myc induction in viral oncogenesis. Interestingly, it has also been demonstrated that miR-155 can suppress TGF-β signaling though targeting SMAD2 and SMAD5 in human diseases (Louafi et al., 2010; Rai et al., 2010). As a viral miR-155 ortholog, the KSHV-encoded miR-K12-11 inhibits TGF-β signaling through down-regulation of SMAD5 (Liu et al., 2012). Collectively, these findings indicate that dysregulation of the TGF-β signaling pathway by miR-155 and its viral orthologs may be a common feature shared by oncogenic herpesviruses (**Figure 2**). Further investigations are required to determine whether there are pathways other than those involving miR-M4-5p that contribute to viral pathogenesis and/or tumorigenesis.

## Conclusion and Prospective

Herpesvirus-encoded miRNAs play important regulatory roles in both active and latent stages of virus infection, although the molecular mechanisms controlling these activities are largely unexplored. The critical viral miR-155 orthologs, miR-M4-5p and miR-K12-11, share a set of common mRNA targets, suggesting the common viral characteristics of mimicking host miRNAs and aberrant regulation of viral miRNAs may be important in herpesvirus life cycle control, immune evasion, pathogenesis, and even tumorigenesis. The rescue of the regulatory role of miR-M4-5p by miR-155 *in vivo* by viral genomic locus replacement proves that the two molecules are authentic functional orthologs. Thus, the shared seed region and common set of target mRNAs of miR-M4-5p, miR-K12-11, and miR-155 have facilitated the characterization of miRNA phenotypes in relation to viral tumorigenesis, as in MD. The development of transcriptome-wide identification of miRNA targets has helped to establish the genomic landscape of GaHV2 miRNA functions. However, results generated using the MSB-1 cell line should be treated with caution, as it is a transformed tumor cell line that is simultaneously infected by GaHV2 and GaHV3. Hence, the existence of GaHV3 miRNAs may interfere with the identification of bona fide mRNA targets of GaHV2 miRNAs. Further mRNA target screening and identification should use MD tumor cells, or cell lines only infected by GaHV2. Overall, the next challenge will be to integrate these findings into a better understanding of how the interference mediated by these miRNAs regulates herpesvirus life cycles and their roles in tumorigenesis.

## Author Contributions

GZ, AS, and MT overview the publications and wrote the manuscript. GZ and JL revised and approved its final version.

## Conflict of Interest Statement

The authors declare that the research was conducted in the absence of any commercial or financial relationships that could be construed as a potential conflict of interest.

## Footnotes

1 https://talk.ictvonline.org/taxonomy/
